# Automated assessment of biological database assertions using the scientific literature

**DOI:** 10.1186/s12859-019-2801-x

**Published:** 2019-04-29

**Authors:** Mohamed Reda Bouadjenek, Justin Zobel, Karin Verspoor

**Affiliations:** 1 Department of Mechanical & Industrial Engineering, University of Toronto, Toronto, M5S 3G8 Canada; 2 School of Computing and Information Systems, University of Melbourne, Melbourne, 3010 Australia

**Keywords:** Data Analysis, Data Quality, Biological Databases, Data Cleansing

## Abstract

**Background:**

The large biological databases such as GenBank contain vast numbers of records, the content of which is substantively based on external resources, including published literature. Manual curation is used to establish whether the literature and the records are indeed consistent. We explore in this paper an automated method for assessing the consistency of biological assertions, to assist biocurators, which we call BARC, Biocuration tool for Assessment of Relation Consistency. In this method a biological assertion is represented as a relation between two objects (for example, a gene and a disease); we then use our novel set-based relevance algorithm SaBRA to retrieve pertinent literature, and apply a classifier to estimate the likelihood that this relation (assertion) is correct.

**Results:**

Our experiments on assessing gene–disease relations and protein–protein interactions using the PubMed Central collection show that BARC can be effective at assisting curators to perform data cleansing. Specifically, the results obtained showed that BARC substantially outperforms the best baselines, with an improvement of F-measure of 3.5% and 13%, respectively, on gene-disease relations and protein-protein interactions. We have additionally carried out a feature analysis that showed that all feature types are informative, as are all fields of the documents.

**Conclusions:**

BARC provides a clear benefit for the biocuration community, as there are no prior automated tools for identifying inconsistent assertions in large-scale biological databases.

## Background

The large biological databases are a foundational, critical resource in both biomedical research and, increasingly, clinical health practice. These databases, typified by GenBank^1^ and UniProt,^2^ represent our collective knowledge of DNA and RNA sequences, genes, proteins, and other kinds of biological entities. The main databases currently contain hundreds of millions of records, each directly or indirectly based on scientific literature or material produced by a reputable laboratory. Each record is contributed by an individual research team, or is derived indirectly from such a contribution, and thus the contents of these databases represent decades of manual effort by the global biomedical community. The databases are used by researchers to infer biological properties of organisms, and by clinicians in disease diagnosis and genetic assessment of health risk [1].

Manual *biocuration* is used with some of the databases to ensure that their contents are correct [2]. Biocuration consists of organizing, integrating, and annotating biological data, with the primary aim of ensuring that the data is reliably retrievable. Specifically, a biocurator derives facts and assertions about biological data, and then verifies their consistency in relevant publications. PubMed^3^ [3], as the primary index of biomedical research publications, is typically consulted for this purpose.

For example, given a database record with the assertion “*the BRCA gene is involved in Alzheimer’s disease*”, a biocurator may search for articles that support or deny that assertion, typically via a PubMed keyword search, and then manually review the articles to confirm the information. Biocuration is, therefore, time-consuming and expensive [4, 5]; curation of a single protein may take up to a week and requires considerable human investment both in terms of knowledge and effort [6].

**Fig. 1:**
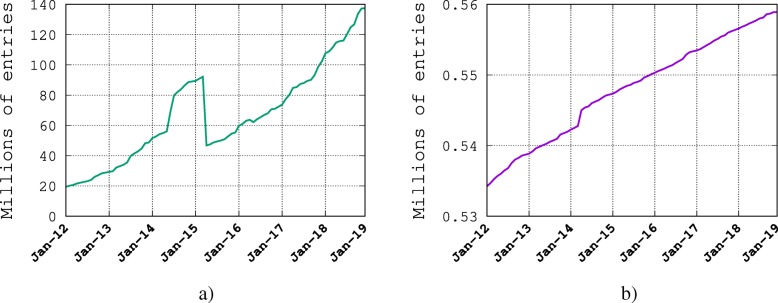
Growth of the number of sequences in UniProt databases. The green and pink lines shows the growth in UniProtKB for TrEMBL and Swiss-Prot respectively entries from January 2012 to January of 2019. The sharp drop in TrEMBL entries corresponds to a proteome redundancy minimization procedure implemented in March 2015 [5]. **a** Growth of TrEMBL. **b** Growth of Swiss-Prot

However, biological databases such as GenBank or UniProt contain hundreds of millions of uncurated nucleic acid and protein sequence records [7], which suffer from a large range of data quality issues including errors, discrepancies, redundancies, ambiguities, and incompleteness [8, 9]. Exhaustive curation on this scale is utterly infeasible; most error detection occurs when submitters re-examine their own records, or occasionally when reported by a user, but it is likely that the rate of error detection is low. Figure 1 illustrates the growth of the curated database UniProtKB/Swiss-Prot against the growth of the uncurated database UniProtKB/TrEMBL (which now contains roughly 89M records). Given the huge gap shown in Fig. 1, it is clear that automated and semi-automated error-detection methods are needed to help and assist biocurators to provide reliable biological data to the research community [10, 11].

In this work, we seek to use the literature to develop an automated method for assessing the consistency of biological assertions. This research builds on our previous work, in which we used the scientific literature to detect biological sequences that may be incorrect [12], to detect literature-inconsistent sequences [13], and to identify biological sequence types [14]. In our previous work on data quality in biological databases, we formalized the quality problem as a characteristic of queries (derived from record definitions); in the discipline of information retrieval there are metrics for estimating query quality. In contrast, in this work we consider the consistency of biological assertions. Previously, we formalized the problem as a pure information retrieval problem, whereas here we also consider linguistic features.

**Fig. 2:**
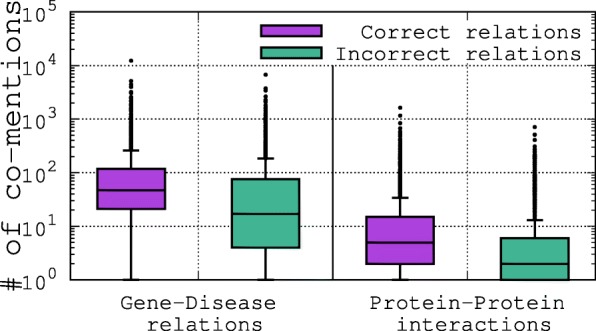
Distribution of co-mention frequencies for *in/correct* relations described in “Experimental data” section. It is apparent that even when two entities are not known to have a valid relationship (an *incorrect* relation), these entities may often be mentioned together in a text (co-mentioned)

To demonstrate the scale of the challenge we address in this paper, consider Fig. 2, which shows the distribution of literature co-mentions (co-occurrences) of correct or incorrect *gene–disease* relations and correct or incorrect *protein–protein* interactions, where correctness is determined based on human-curated relational data (described further in “Experimental data” section). For example, a *gene–disease* relation represents an assertion of the form *Gene–Relation–Disease*, where *Relation* is a predicate representing the relationship between the *gene* and the *disease*, such as “causes”, “involved in”, or “related to”. This analysis shows that, despite the fact that entities that are arguments of correct relations tend to be co-mentioned more often than those in incorrect relations, simplistic filtering methods based on a frequency threshold are unlikely to be effective at distinguishing correct from incorrect relations. Moreover, for many incorrect relations, the entities that are arguments of the relation are often mentioned together in a text (co-mentioned) despite not being formally related. Therefore, more sophisticated techniques are needed to address this problem.

We have developed *BARC*, a Biocuration tool for Assessment of Relation Consistency.^4^ In BARC, a biological assertion is represented as a relation between two entities (such as a gene and a disease), and is assessed in a three-step process. First, for a given pair of objects (*object*_1_,*object*_2_) involved in a relation, BARC retrieves a subset of documents that are relevant to that relation using *SaBRA*, a document ranking algorithm we have developed. The algorithm is based on the notion of the *relevant set* rather than an individual *relevant document*. Second, the set of retrieved documents is aggregated to enable extraction of relevant features to characterise the relation. Third, BARC uses a classifier to estimate the likelihood that this assertion is correct. The contributions of this paper are as follows:

1. We develop a method that uses the scientific literature to estimate the likelihood of correctness of biological assertions.
2. We propose and evaluate SaBRA, a ranking algorithm for retrieving document *sets* rather than individual documents.
3. We present an experimental evaluation using assertions representing gene–disease relations and protein–protein interactions on the PubMed Central Collection, where BARC achieved, respectively, 89% and 79% of accuracy.

Our results show that BARC, which compiles and integrates the methods and algorithms developed in this paper, outperforms plausible baselines across all metrics, on a dataset of several thousand assertions evaluated against a repository of over one million full-text publications.

## Problem definition

Biocuration can be defined as the transformation of biological data into an organized form [2]. To achieve this, a biocurator typically manually reviews published literature to identify assertions related to entities of interest with the goal of enriching a curated database, such as UniProtKB/Swiss-Prot. However, there are also large databases of uncurated information, such as UniProtKB/TrEMBL, and biocurators would also need to check the assertions within these databases for their veracity, again with respect to the literature. Biological assertions that biocurators check are of various types. For example, they include:

- **Genotype-phenotype relations (OMIM db):** these include assertions about gene–disease relations or mutation-disease or gene-disease relations [15]. A biocurator will then have to answer questions such as: “*is the BRCA gene involved in Alzheimer’s disease?*”
- **Functional residue in protein (Catalytic Site Atlas or BindingMOAD databases):** these include assertions about sub-sequences being a functional residue of a given protein [16, 17]. An example question is: “*is Tyr247 a functional residue in cyclic adenosine monophosphate (cAMP)-dependent protein kinase (PKA)?*”
- **Protein-protein interaction (BioGRID db):** these include assertions about interactions between proteins. An example question is: “*is the protein phosphatase PPH3 related to the protein PP2A?*” [18].
- **Drug-treats-disease (PharmGKB database):** which includes assertions about a drug being a treatment of a disease. An example question is: “*can Tamoxifen be used to treat Atopy*^5^?” [19].
- **Drug-causes-disease (CTD database):** these include assertions about drugs causing a disease [20]. An example question is: “*can paracetamol induce liver disease?*”.

The biocuration task is time-consuming and requires a considerable investment in terms of knowledge and human effort. A supportive tool has the potential to save significant time and effort.

In this work, we focus on the analysis of only two type of relations, namely gene-disease and gene-gene relations. We leave the analysis of other relations to future work.

We propose to represent and model a *relation*, defined as follows:

### **Definition 1**

Let *O*_1_ and *O*_2_ be respectively a set of objects of type 1 and a set of objects of type 2. A *relationR* is a triplet of the form (*object*_1_,*predicate*,*object*_2_), where *object*_1_∈*O*_1_ and *object*_2_∈*O*_2_ are entities that have some relationship between them as indicated by the predicate.

For example, the assertion “*The gene CFTR causes the disease cystic fibrosis*” is represented by the gene–disease relation *R*:

*R*=(*CFTR*,*causes*,*Cystic fibrosis*) where *CFTR* is of type gene and *Cystic fibrosis* is of type disease.

Note that the transformation of an assertion from its linguistic declarative form to the relational form is undertaken by the biocurator, and is out of the scope of this paper.

We formally define the problem we study as follows. Given

- A collection of documents that represents the domain literature knowledge *D*=<*d*_1_,*d*_2_,…,*d*_*k*_>;
- A set of *n* relation types *T*={*T*_1_,*T*_2_,...,*T*_*n*_}, where *R*_*m*_∈*T*_*n*_ defines a relation between two objects that holds in the context of the assertion type *T*_*n*_;
- A set of annotated relations $R_{T_{n}}$ for a particular assertion type *T*_*n*_ such as $R_{T_{n}}=<(R_{1},y_{1}),(R_{2},y_{2}),\ldots,(R_{m},y_{m})>$, where *R*_*m*_∈*T*_*n*_ and *y*_*m*_∈{*correct*,*incorrect*};

we aim to classify a new and unseen relation *R*_*p*_ of type *T*_*l*_ as being *correct* or *incorrect* given the domain literature knowledge *D*. In other words, we seek support for that assertion in the scientific literature. The resulting tool described in the next sections is expected to be used at curation time.

## Method

**Fig. 3:**
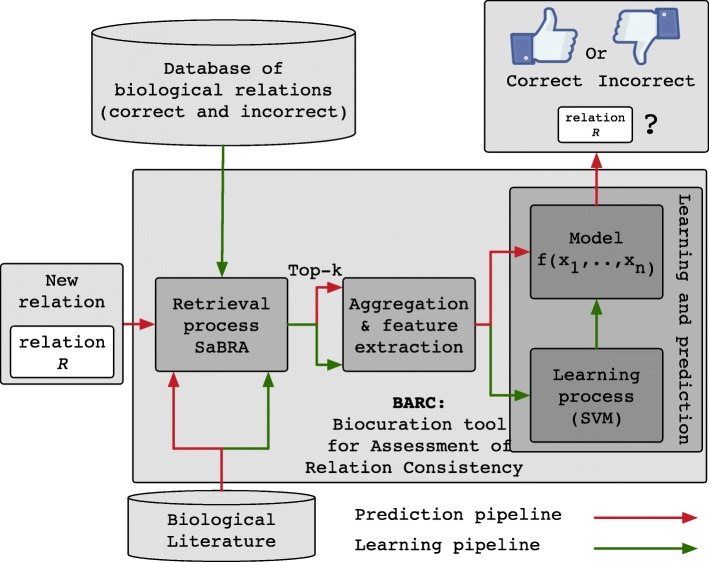
Architecture overview of BARC

Figure 3 describes the logical architecture of our tool, *BARC*, a Biocuration tool for Assessment of Relation Consistency. The method embodied in BARC uses machine learning, and thus relies on a two-step process of learning and predicting. At the core of BARC are three components that we now describe.

**Retrieval (“**SaBRA**” section):** This component handles the tasks of processing a relation and collecting a subset of the documents that are used to assess the validity of that assertion. The inputs of this component are a relation and the document index built for the search task. The internal search and ranking algorithm implemented by this component is described later. Indexing of the collection is out of the scope of this paper, but is briefly reviewed in “Experimental data” section.

**Aggregation & feature extraction (“**Relation consistency features**” section):** This component takes as an input the set of documents returned by the *retrieval component* and is responsible for the main task of aggregating this set of documents to allow extraction of relevant features. Three kinds of features are then computed and produced, (i) *inferred relation words*, (ii) *co-mention based*, and (iii) *context similarity-based*. These features are discussed in “Relation consistency features” section.

**Learning and prediction (“**Supervised learning algorithm**” section):** This component is the machine-learning core of BARC. It takes as input feature vectors, each representing a particular relation. These feature vectors are processed by two sub-components depending on the task: the *learning pipeline* and the *prediction pipeline*. The *learning pipeline* is responsible for the creation of a model, which is then used by the *prediction pipeline* to classify unseen assertions as being *correct* or *incorrect*.

## *SaBRA* for ranking documents

In typical information retrieval applications, the objective is to return lists of documents from a given document collection, ranked by their relevancy to a user’s query. In the case of BARC, the documents being retrieved are not intended for individual consideration by a user, but rather with the purpose of aggregating them for feature extraction for our classification task. Hence, given a relation *R*=(*object*_1_,*predicate*,*object*_2_), we need to select documents that mention both *object*_1_ and *object*_2_ at a suitable ratio, to allow accurate computation of features; a set of documents that is biased towards documents containing either *object*_1_ or *object*_2_ will result in low-quality features. Standard IR models such as the vector-space model TF-IDF [21] or the probabilistic model BM25 [22] are not designed to satisfy this constraint.


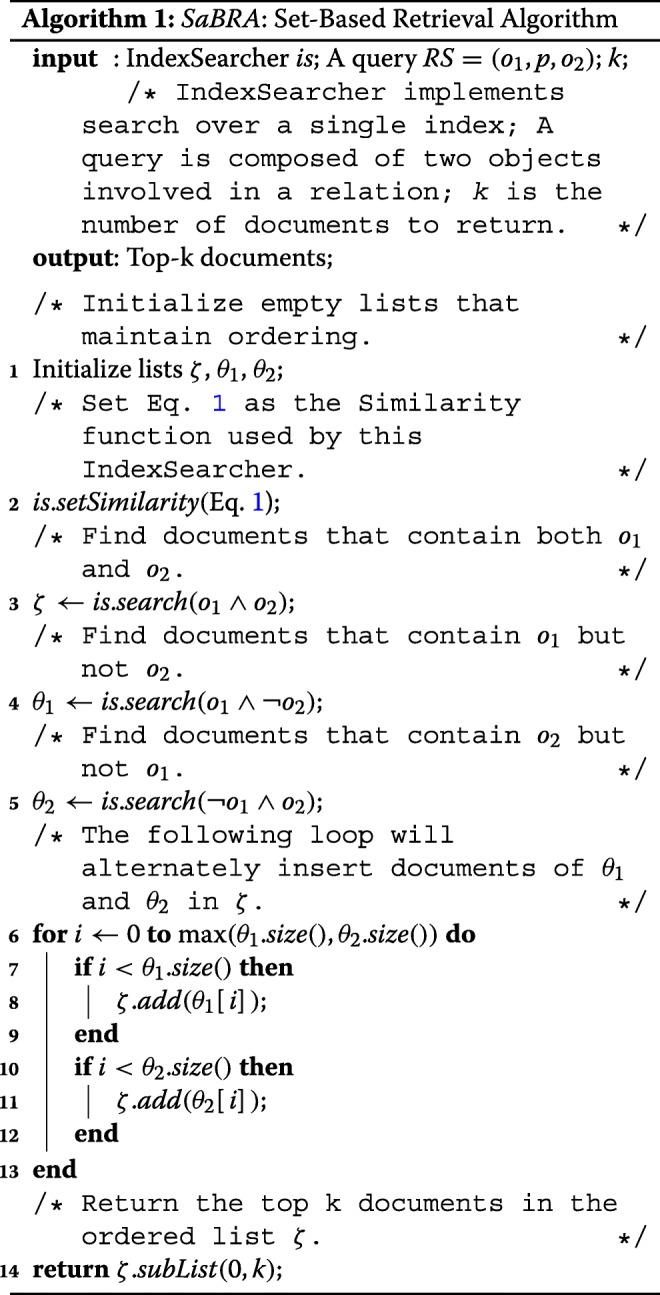


Given the settings and the problem discussed above, we developed *SaBRA*, a Set-Based Retrieval Algorithm, as summarized in Algorithm 1. In brief, SaBRA is designed to guarantee the presence of a reasonable ratio of mentions for *object*_1_ and *object*_2_ in the top *k* documents retrieved, to extract features for a relation *R*=(*object*_1_,*predicate*,*object*_2_).

SaBRA takes in input an IndexSearcher that implements search over a single index, a query in the form of a relation assertion *R*=(*o*_1_,*p*,*o*_2_), where *k* the number of documents to return (choice of *k* is examined later). First, SaBRA initializes three empty ordered lists *ζ*, *θ*_1_, and *θ*_2_ (line 1; these sets are explained below), and sets the similarity function the IndexSearch used (line 2). Given a relation *R* and a document *d*, the scoring function used to compute the similarity is the following:

1 $$ {\begin{aligned} {}{f(R,d)=\frac{\log[1+count(o_{1},d)]}{(1-b)+b\times|d|_{o_{1}}}+\frac{\log[1+count(o_{2},d)]}{(1-b)+b\times|d|_{o_{2}}}} \end{aligned}}  $$

where *count*(*o*,*d*) is the number of time *o* occurs in *d*, the value |*d*|_*o*_ is the number of mentions of objects of the same type as *o* in document *d*, and *b* is a weighting parameter empirically determined and set to 0.75 in our experiments. The intuition behind the denominator for the two terms is to penalize documents that mention other objects of the same type as *o* [23], such as other genes or diseases other than those involved in the relational assertion being processed. The numerator acts provides term frequency weighting.

Next, SaBRA retrieves: (i) documents that mention both *o*_1_ and *o*_2_ in the ordered list *ζ* (line 3), (ii) documents that mention *o*_1_ but not *o*_2_ in the ordered list *θ*_1_ (line 4), and (iii) documents that mention *o*_2_ but not *o*_1_ in the ordered list *θ*_2_ (line 5). Then, SaBRA alternately inserts documents of *θ*_1_ and *θ*_2_ at the end of *ζ* (lines 6 to 13). Documents in *ζ* are considered to be more significant as they contain documents that mention and may link the two objects involved in the relation being processed. Finally, SaBRA returns the top-*k* documents of the ordered list *ζ*(line 14).

## Relation consistency features

We now explain the features that BARC extracts from the set of documents retrieved by its retrieval component through SaBRA.

### Inferred relation word features

Given a correct relation *R*=(*object*_1_,*predicate*,*object*_2_), we expect that *object*_1_ and *object*_2_ will co-occur in sentences in the scientific literature (co-mentions), and that words expressing the *predicate* of the target relation between them will also occur in those sentences. This assumption is commonly adopted in resources for and approaches to relation extraction from the literature, such as in the HPRD corpus [24, 25]. Following this intuition, we have automatically analyzed correct relations of the training set described in “Experimental data” section to extract words that occur in all sentences where the two objects of each correct relation are co-mentioned. Tables 1 and 2 show the top 5 co-occurring words for the correct relations we have considered, their frequencies across all sentences, and example sentences in which the words co-occur with the two objects involved in a relevant relation in the data set. For example, the most common word that occurs with gene–disease pairs is the word “*mutation*”; it may suggest the relation between them. These words can be considered to represent the relation predicate *R*. Hence, these inferred relation word features can capture the predicate.

**Table 1:** Examples of the top 5 words that occur in sentences where genes and diseases of the relations described in “Experimental data” section also occur

	Term	Frequency	Example
1	Mutation	26,020	**Mutations** of the **PLEKHM1** gene have been identified as the cause of the **osteopetrotic** ia/ia rat. [PMID:22073305]
2	Express	5,738	**RAD51** was reported to have a significantly increased **expression** in **breast cancer**. [PMID: 23977219]
3	Result	5,151	**HTTASresults** in homozygous **HD** cells. [PMID: 25928884]
4	Activate	4,454	**FGFR2** has been shown to **activate** signal transduction leading to transformation in **breast cancer**. [PMID: 25333473]
5	Risk	4,423	**RNF213** was recently identified as a major genetic **risk** factor for **moyamoya disease**. [PMID: 25964206 ]

These words can be seen as approximating the semantics of the predicates linking the genes and the diseases. The PubMed ID (PMID) of the source article for each example is provided in brackets

Words in bold represent entities involved in an assertion, i.e., the entities and the predicate

**Table 2:** Examples of the top 5 words that occur in sentences where two proteins that interact also occur

	Term	Frequency	Example
1	Interact	33,128	As reported previously we also confirmed **PHD2 interaction** with **FKBP38**. [PMID: 21559462]
2	Activ	30,241	Another known protein that modulates **PHD2 activity** is **FKBP38**. [PMID: 20178464 ]
3	Express	29,863	BMP2 may promote **PHD2** stabilization by down-modulating **FKBP38 expression**. [PMID: 19587783]
4	Bind	29,468	**mIL-5** showed similar, high-affinity **binding** profiles to both **gpIL-5r** and **hIL-5r**. [PMID: 11132776]
5	Regulator	15,939	As a **regulator** of **JNK**, **POSH** is mainly implicated in the activation of apoptosis. [PMID: 17420289]

These words can be seen as approximating the semantics of the predicates linking the two proteins

Words in bold represent entities involved in an assertion, i.e., the entities and the predicate

For each relation type we have curated a list of the top 100 co-occurring words as described above. Then, for each relation *R*=(*object*_1_,*predicate*,*object*_2_) of type *T*, a feature vector is defined with values representing the frequency of appearance of that word in sentences where *object*_1_ and *object*_2_ occur. Hence, our model will inherently succeed in capturing different predicates between the same pair of objects. We separately consider the three different fields of the documents (title, abstract and body). In total, we obtain 300 word-level features for these inferred relation words.

### Co-mention-based features

Following the intuition that for a correct relation *R*=(*object*_1_,*predicate*,*object*_2_), *object*_1_ and *object*_2_ should occur in the same sentences of the scientific literature, we have tested several similarity measures that compute how often they occur in the same sentences. These co-mention similarity measures – Dice, Jaccard, Overlap, and Cosine [26] – are computed as defined in Table 3. These similarity measures are also computed while considering separately the title, the abstract, and the body of the documents, giving a total of 12 features.

**Table 3:** Co-mention similarity measures summarization

Dice	$D(o_{1},o_{2})=2\times \frac {\mid Sentences(o_{1})\cap Sentences(o_{2})\mid }{\mid Sentences(o_{1})\mid +\mid Sentences(o_{2})\mid }$
Jaccard	$J(o_{1},o_{2})=\frac {\mid Sentences(o_{1})\cap Sentences(o_{2})\mid }{\mid Sentences(o_{1})\cup Sentences(o_{2})\mid }$
Overlap	$O(o_{1},o_{2})=\frac {\mid Sentences(o_{1})\cap Sentences(o_{2})\mid }{min(Sentences(o_{1}),Sentences(o_{2}))}$
Cosine	$Cos(o_{1},o_{2})=\frac {\mid Sentences(o_{1})\cap Sentences(o_{2})\mid }{\sqrt {\mid Sentences(o_{1})\mid \times \mid Sentences(o_{2})\mid }}$

The function *Sentences*(*o*) returns from a set of documents those sentences where the object *o* occurs

### Context similarity-based features

Given a relation *R*=(*object*_1_,*predicate*,*object*_2_), the strength of the link associating the two objects can be estimated in a set of documents by evaluating the similarities of their context mentions in the text, following the intuition that two objects tend to be highly related if they share similar contexts. Hence, to evaluate the support of a given relation *R* that associates two objects given a set of documents, we define a context similarity matrix as follows:

#### **Definition 2**

A context similarity matrix $\phantom {\dot {i}\!}M^{(o_{1},o_{2})}$ associated with a set of documents *D* is a matrix that reports the similarity between the context of two objects *o*_1_ and *o*_2_ such that each entry (*i*,*j*) estimates the similarity between the *i*^*th*^ mention of the object *o*_1_ in the set *D*, and the *j*^*th*^ occurrence of the object *o*_2_ in the set *D*.

**Fig. 4:**
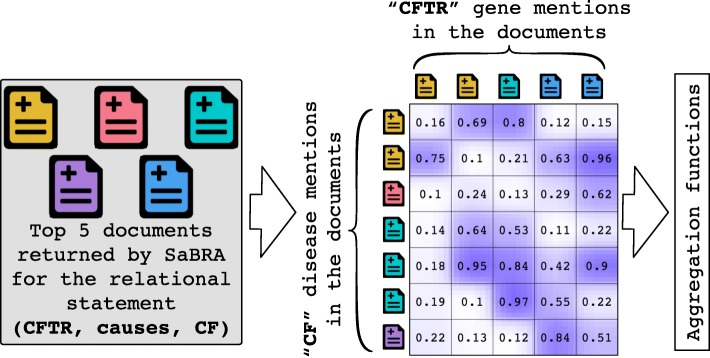
Toy example for building the context similarity matrix of the relational statement (*CFTR*, *causes*, *CF*) from the top 5 documents returned by SaBRA. Similarities are computed between the contexts of each occurrence of the two entities in the top documents. Aggregation values are then computed based on the obtained matrix to construct a feature vector

Figure 4 shows the context similarity matrix for two objects, the gene “CFTR” and the disease “Cystic fibrosis (CF)”. This matrix indicates, for example, that the context of the first occurrence of the gene “CFTR” in the first returned document has a similarity of 0.16 with the context of the first occurrence of the disease CF in the same document. Similarly, the context of the first occurrence of the gene in that document has a similarity of 0.22 with the context of the first occurrence of the disease CF in the fourth returned document. Essentially, the concept–concept matrix captures the lexical similarity of the different occurrences of two objects in the documents returned by SaBRA. Once this matrix is built, we can calculate aggregate values based on the sum, standard deviation, minimum, maximum, arithmetic mean, geometric mean, harmonic mean, and coefficient of variation of all computed similarities. These aggregated values can be used as summaries of the link strength of the two objects.

In the following, we first describe how we estimate the context distribution of a given object (word) in an article. Then, we describe different probabilistic-based metrics that we use to estimate the similarity between the contexts of the different occurrences of two objects.

Based on the method proposed by Wang and Zhai [27], who defined the context of a term based on a left and a right window size, we empirically found (results not shown) that the context of a word is best defined using sentence boundaries, as follows:

#### **Definition 3**

The context (*C*) of a document term *w* is the set of words that occur in the same sentence than *w*.

For example, in the sentence *“Alice exchanged encrypted messages with Bob”*, we say that the words *“Alice”*, *“encrypted”*, *“messages”*, *“with”*, and *“Bob”* are in the context *C* of the word *“exchanged”*.

Let *C*(*w*) denotes the set of words that are in the *C* context of *w* and *count*(*a*,*C*(*w*)) denotes the number of times that the word *a* occurs in the context *C* of *w*. Given a term *w*, the probability distribution of its context words using Dirichlet prior smoothing [28] is given by:

2 $$ \tilde{P}_{C}(a|w)=\frac{count(a,C(w))+\mu P(a|\theta)}{{\sum\limits_{i\in C(w)}count(i,C(w))}+\mu}  $$

where *P*(*a*|*θ*) is the probability of the word *a* in the entire collection and *μ* is the Dirichlet prior parameter, which was empirically defined and set to 1500 in all of our experiments. Analogously, we define the double conditionalprobability distribution over the contexts of two words *w*_1_ and *w*_2_ as:

3 $$ \begin{aligned} \begin{array}{l} {}{\tilde{P}(c|w_{1},w_{2})=\frac{{count(c,C(w_{1}))+count(c,C(w_{2}))}+\mu P(c|\theta)}{{\sum\limits_{i\in C(w_{1})}\sum\limits_{j\in C(w_{2})}\left({count(i,C(w_{1}))+count(j,C(w_{2}))}\right)}+\mu}} \end{array} \end{aligned}  $$

The similarity model is used to capture whether two objects of a relational statement are sharing the same context. The idea is that objects that occur in similar contexts are related to each another, and thus are highly correlated. In the following, we describe several similarity measures we used; these are fully described elsewhere [26].

**Overlap:***Overlap similarity* is a similarity measure that measures the overlap between two sets and is defined as the size of the intersection divided by the smaller of the size of the two sets. In a probabilistic distributional case, it is defined as:

4 $$ {{\begin{aligned} {}{O(w_{1},w_{2})=\frac{{\sum\limits_{c\in C(w_{1})\cap C(w_{2})}\log\tilde{P}(c|w_{1},w_{2})}}{max\left({\sum\limits_{a\in C(w_{1})}\log\tilde{P}(a|w_{1})},{\sum\limits_{b\in C(w_{2})}\log\tilde{P}(b|w_{2})}\right)}} \end{aligned}}}  $$

where $\tilde {P}(a|w_{1})$ and $\tilde {P}(b|w_{2})$ are defined in Eq. 2, and the joint probability $\tilde {P}(c|w_{1},w_{2})$ is defined as in Eq. 3.

**Matching:** The *matching similarity*measure is defined as:

5 $$ M(w_{1},w_{2})={\sum\limits_{c\in C(w_{1})\cap C(w_{2})}\log\tilde{P}(c|w_{1},w_{2})}  $$

**Jaccard:** The *Jaccardsimilarity* is a statistic metric used for comparing the similarity and diversity of sample sets. It is defined as the size of the intersection divided by the size of the union of the sample sets. In a probabilistic distributional case, it is defined as:

6 $$ J(w_{1},w_{2})=\frac{{\sum\limits_{c\in C(w_{1})\cap C(w_{2})}\log\tilde{P}(c|w_{1},w_{2})}}{{\sum\limits_{c\in C(w_{1})\cup C(w_{2})}\log\tilde{P}(c|w_{1},w_{2})}}  $$

**Dice:** The *Dice similarity* measure is defined analogously to the harmonic mean between two sets. It considered as a semi-metric since it doesn’t satisfy the triangle inequality property. In a probabilistic distributional case, it is defined as follows:

7 $$ D(w_{1},w_{2})=\frac{2\times{\sum\limits_{c\in C(w_{1})\cap C(w_{2})}\log\tilde{P}(c|w_{1},w_{2})}}{{\sum\limits_{a\in C(w_{1})}\log\tilde{P}(a|w_{1})}+{\sum\limits_{b\in C(w_{2})}\log\tilde{P}(b|w_{2})}}  $$

**Cosine:** The *cosine similarity* measure is defined as the inner product space that measures the cosine of the angle between vectors. In a probabilistic distributional case, it is defined as follows:

8 $$ {{\begin{aligned} {}Cos(w_{1},w_{2})=\frac{{\sum\limits_{c\in C(w_{1})\cap C(w_{2})}\log\tilde{P}(c|w_{1})}\log\tilde{P}(c|w_{2})}{\sqrt{{\sum\limits_{a\in C(w_{1})}\left[\log\tilde{P}(a|w_{1})\right]^{2}}}\times\sqrt{{\sum\limits_{b\in C(w_{2})}\left[\log\tilde{P}(b|w_{2})\right]^{2}}}} \end{aligned}}}  $$

We apply these five similarity measures to build context similarity matrices, with each column constructed separately based on the title, the abstract, and the body of the returned documents. Once these matrices are built, we calculate for each matrix aggregation values based on the sum, standard deviation, minimum, maximum, arithmetic mean, geometric mean, harmonic mean, and coefficient of variation. In total, we have defined 120 context similarity-based features.

### Summary

In summary, we have defined 300 word-level features for the inferred relation words, 12 co-mention-based features, and 120 context similarity-based features. Therefore, for each relational statement we have a total of 432 feature values, which can be represented as a feature vector *x*_*m*_=[*x*_*m*1_,*x*_*m*2_,…,*x*_*m*432_].

## Supervised learning algorithm

Given as input a set of features for each relation to assess, our goal is to combine these inputs to produce a value indicating whether this relation is correct or incorrect given the scientific literature. To accomplish this, we use SVMs [29], one of the most widely-used and effective classification algorithms.

Each relation *R*_*m*_ is represented by its vector of 432 features *x*_*m*_=[*x*_*m*1_,*x*_*m*2_,…,*x*_*m*432_] and its associated label *y*_*m*_∈{*correct*,*incorrect*}. We used the SVM implementation available in the LibSVM package [30]. Both Linear and RBF kernels were considered in our experiments. The regularization parameter *C* (the trade-off between training error and margin), and the gamma parameter of the RBF kernel are selected from a search within the discrete sets {10^−5^,10^−3^,…,10^13^,10^15^}, and {10^−15^,10^−13^,…,10^1^,10^3^}, respectively. Each algorithm is assessed using a nested cross validation approach, which effectively uses a series of 10 train–test set splits. The inner loop is responsible for model selection and hyperparameter tuning (similar to a validation set), while the outer loop is for error estimation (test set), thus, reducing the bias.

In the inner loop, the score is approximately maximized by fitting a model that selects hyper-parameters using 10-fold cross-validation on each training set. In the outer loop, efficiency scores are estimated by averaging test set scores over the 10 dataset splits. Although the differences were not substantial, initial experiments with the best RBF kernel parameters performed slightly better than the best linear kernel parameters for the majority of the validation experiments. Unless otherwise noted, all presented results were obtained using an RBF kernel, with *C* and gamma set to the values that provide the best accuracy.

## Experimental data

We first describe the collection of documents we have used for the evaluation, then describe the two types of relations we have considered.

**Literature:** We used the PubMed Central Open Access collection^6^ (OA), which is a free full-text archive of biomedical and life sciences journal literature at the US National Institutes of Health’s National Library of Medicine. The release of PMC OA we used contains approximately 1.13 million articles, which are provided in an XML format with specific fields corresponding to each section or subsection in the article. We indexed the collection based on genes/proteins and diseases that were detected in the literature while focusing on human species. To identify genes or proteins in the documents we used GNormPlus [31]. (Note that the namespace for genes and proteins overlaps significantly and this tool does not distinguish between genes or proteins.) GNormPlus has been reported to have precision and recall of 87.1% and 86.4%, respectively, on the BioCreative II GN test set. To identify disease mentions in the text, we used DNorm [32], a tool reported to have precision and recall of 80.3% and 76.3%, respectively, on the subset of the NCBI disease corpus. The collection of documents is indexed at a concept level rather than on a word level, in that synonyms, short names, and long names of the same gene or disease are all mapped and indexed as the same concept. Also, each component of each article (title, abstract, body) is indexed separately, so that different sections can be used and queried separately to compute the features [33].**Gene-disease relations:** The first type of relation that we used to evaluate BARC is etiology of disease, that is, the gene-causes-disease relation. To collect correct gene–disease relations (positive examples), we used a curated dataset from Genetics Home Reference provided by the Jensen Lab [34]^7^. Note that we kept only relations for which GNormPlus and DNorm identified at least a single gene and disease respectively. To build a test set of incorrect relations (negative examples), we used the Comparative Toxicogenomics Database (CTD), which contains both curated and inferred gene–disease associations [20].

The process for generating negative examples was as follows: (i) We determined the set of documents from which the CTD dataset has been built, using all PubMed article identifiers referenced in the database for any relation. (ii) We automatically extracted all sentences in which a gene and a disease are co-mentioned (co-occur) that appear in the set of documents, and identified the unique set of gene–disease pairs across these sentences. (iii) We removed all gene–disease pairs that are known to be valid, due to being in the curated CTD dataset. (iv) We manually reviewed the remaining gene–disease pairs, and removed all pairs for which evidence could be identified that suggested a valid (correct) gene–disease relation (10% of the pairs were removed at this step by reviewing about 5–10 documents for each relation). The remaining pairs are our set of negative examples. We consider this data set to consist of reliably incorrect relations (reliable negatives), based on the assumption that each article that is completely curated, that is, that any relevant gene–disease relationship in the article is identified. This is consistent with the article-level curation that is performed by the CTD biocurators [20].

**Protein-protein interactions:** The second kind of relation we used to evaluate BARC is protein–protein interactions (PPIs). We used the dataset provided by BioGRID as the set of correct relations [35].^8^ We kept only associations for which the curated documents are in our collection. To build a test set of incorrect relational statements, we proceeded similarly to the previous case, again under the assumption that all documents are exhaustively curated; if the document is in the collection, all relevant relations should have been identified.

We describe our dataset in Table 4. For example, articles cite 6.15 genes on average; the article PMC100320^9^ cites 2040 genes. A gene is cited on average 24.6 times, while the NAT2^10^ is the most cited gene. GNormPlus and DNorm identified respectively roughly 54M genes and 55M diseases in the collection.

Finally, in the experimental evaluation, we consider a total of 1991 gene–disease relations, among which 989 are correct and 1002 are incorrect. On average each mention is in 141.9 documents, with a minimum of 1 and a maximum of 12,296. Similarly, we consider a total of 4,657 protein–protein interactions among which 1758 are correct and 2899 are incorrect. Hence, our test set has reasonable balance.

## Results

Our experiments address the following questions, in the context of the task of classifying whether or not a given relational assertion is supported by the literature:

1. How well does SaBRA perform the task of building a relevant set for feature extraction, compared to other retrieval methods?
2. How well does SaBRA perform on relations with different document support values for the two objects involved in these relations?
3. How does BARC compare with other approaches to the same task?

### Evaluation of *SaBRA*

Given that SaBRA is designed to retrieve documents for a specific task of classification, standard evaluation approaches and metrics of information retrieval are not applicable. Therefore, we chose to evaluate the performance of SaBRA by examining the general performance of the classification task, that is, the performance of BARC. As baselines, we compared SaBRA with two well-known scoring functions: TF-IDF and Okapi BM25. Note that we also use named entities for the retrieval step and that we use these two functions for ranking only.^11^ Specifically, TF-IDF and BM25 are applied in place of lines 6-13 in Algorithm 14, to order the documents previously retrieved on lines 3-5. The performance is assessed using conventional metrics used to evaluate a classifier, namely: precision, recall, accuracy, Receiver Operating Characteristic curve (ROC curve), and Area Under the ROC curve (ROC AUC).

**Fig. 5:**
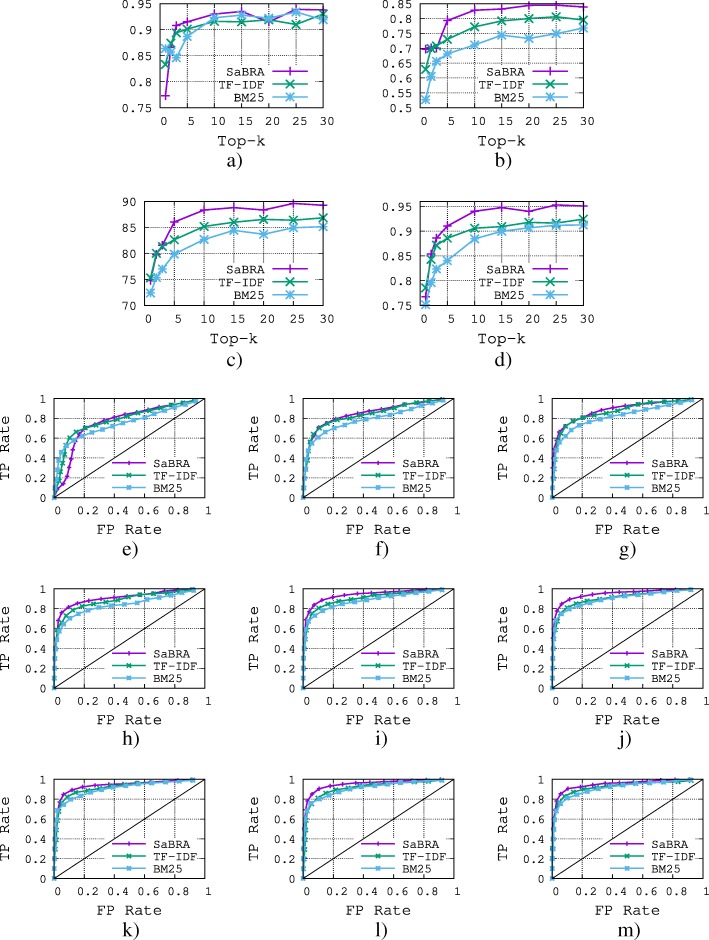
Comparison of *SaBRA* with TF-IDF and BM25 scoring functions using gene-disease relations. **a** Precision for correct statements. **b** Recall for correct statements. **c** Classification Accuracy. **d** ROC AUC. **e** ROC K =1. **f** ROC K =2. **g** ROC K =3. **h** ROC K =5. **i** ROC K =10. **j** ROC K =15. **k** ROC K =20. **l** ROC K =25. **m** ROC K =30

**Fig. 6:**
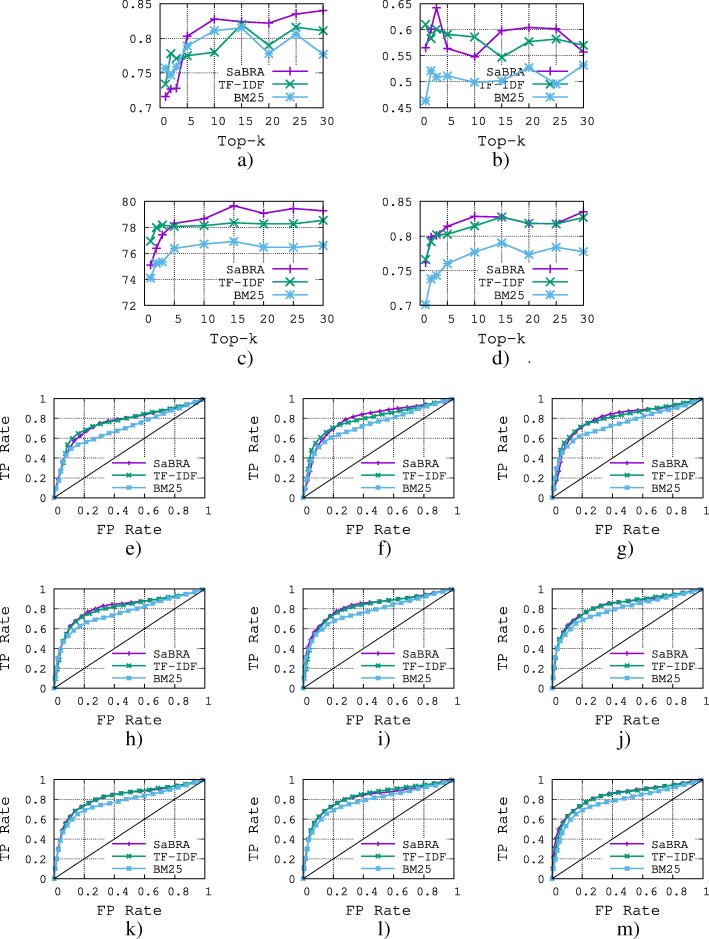
Comparison of *SaBRA* with TF-IDF and BM25 scoring functions using protein-protein interactions. **a** Precision for correct statements. **b** Recall for correct statements. **c** Classification Accuracy. **d** ROC AUC. **e** ROC K =1. **f** ROC K =2. **g** ROC K =3. **h** ROC K =5. **i** ROC K =10. **j** ROC K =15. **k** ROC K =20. **l** ROC K =25. **m** ROC K =30

The results of the comparison are shown in Figs. 5 and 6 for gene–disease relations and protein–protein interactions respectively. We also show results obtained for values of *k*∈{1,2,3,5,10,15,20,25,30}, which is the number of top documents returned by the retrieval algorithms. From the results, we make the following observations.

In general, the method works well. BARC achieves an accuracy of roughly 89% and 79%, respectively, for the gene–disease relations and protein–protein interactions. The higher the value of *k*, the higher the performance of the classification. This is almost certainly due to the fact that the higher the number of aggregated documents, the more likely it is that the features are informative, and thus, the higher the performance of the classification. However, we note that performance is more or less stable above *k*=10 for both gene–disease relations and protein–protein interactions. Considering more documents in both cases results in only marginal improvement.

While the performance obtained when varying *k* on the gene–disease relations is smooth (the performance keeps increasing as *k* increases), the performance while varying k on the protein–protein interactions is noisy. For example, for *k*=3 SaBRA achieved 65% recall, but for *k*=5 the recall dropped to 56%, which means the two documents added are probably irrelevant for building a relevant set of documents. Similar observations can also be made for the two baselines. For almost all values of *k*, SaBRA outperforms the two retrieval baselines BM25 and TF-IDF. While SaBRA clearly outperforms BM25 (roughly 13% for recall, 6% for accuracy, 5% ROC AUC for gene–disease relations), the improvement over TF-IDF is lower. In the next section, we explore how SaBRA performs on different statements with respect to the two retrieval algorithms.

Overall, the performance obtained on the gene–disease relations is higher than that obtained for protein–protein interactions. This is probably because genes and diseases that are related tend to be more often associated in the literature than are proteins that interact; indeed, gene–disease relations attract more attention from the research community. Therefore, there is more sparsity in the protein–protein interactions test set. This is also reported in Table 4, where on average each gene–disease relation has a support of 141.9, whereas each protein–protein interaction relation has a support of 14.1.

### Performance on different relations

Relational statements may be analyzed using the support criteria to identify the most important or frequent relationships. We define the support as a measurement of how frequently the relations appear in the same documents in the literature. Clearly, correct relations with low support values are of weaker association evidence than correct relations with high support values. Analogously, incorrect relations with high support values may be of stronger association evidence than incorrect relations with low support values. Therefore, we perform an evaluation analysis based on relations with different document support values.

**Fig. 7:**
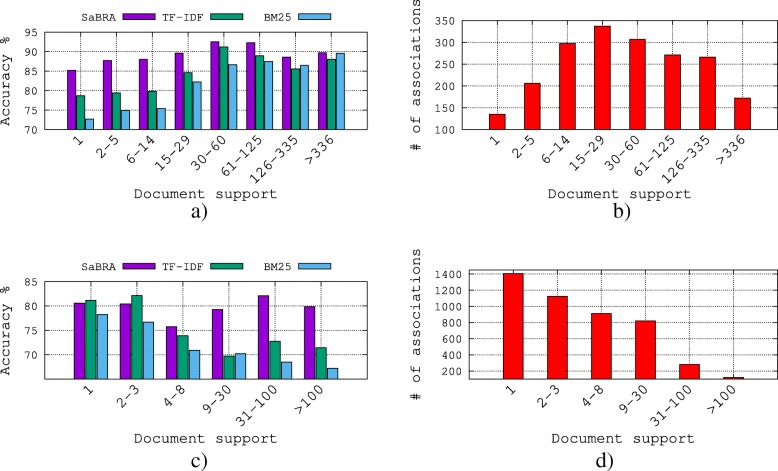
Performance comparison of *SaBRA* on different relations with different document support values for *k*=30. **a** Performance comparison for different document support values on gene-disease relations. **b** Distribution of gene-disease relations. **c** Performance comparison for different document support values on protein-protein interactions. **d** Distribution of protein-protein interactions

In order to evaluate BARC in general and SaBRA in particular on relations with document support values, we first group all the relations based on the document support values in the dataset, and then evaluate the accuracy of different relation groups. Results comparing SaBRA against the other retrieval methods are shown in Fig. 7. For gene–disease relations, we build eight classes: “1”, “2 − 5”, “6 − 14”, “15 − 29”, “30 − 60”, “61 − 125”, “126 − 335”, and “> 335”, denoting the document support values for each group. As for the protein–protein interactions, relations are grouped in 6 classes: : “1”, “2 − 3”, “4 − 8”, “9 − 30”, “31 − 100”, and “> 100”.

Figure 7b and d summarize the distributions of, respectively, the gene–disease relations and the protein–protein interactions according to the groups defined. For example, in Fig. 7b, there are a total of 135 gene–disease relations evaluated in for which the document support values equal to 1. The performance is evaluated in terms of accuracy in Fig. 7a and c. SaBRA generally performs better than the other methods.

The conclusions drawn are different for the two relational statements evaluated. For gene–disease relations with low document support values (“1”), SaBRA performs much better than TF-IDF and BM25, and increases the performance with roughly 10% and 18% respectively. As the document support values increase, the performances of all the algorithms increase and converge, but SaBRA still generates better predictions than the other methods at all levels.

For protein–protein relations with high document support values (“>100”), SaBRA performs much better than TF-IDF and BM25, and increases the performance with roughly 12% and 19% respectively. However, as the document support values decrease, the performance of SaBRA is consistent but the performances of TF-IDF and BM25 increase. TF-IDF is even doing slightly better than SaBRA for relations with document support values in the range “1-3”, yielding an improvement of roughly 1%.

### Classification performance

In the previous sections we focused on the evaluation of SaBRA, the ranking algorithm used in BARC. Here, we evaluate BARC itself with respect to straightforward baselines (noting that there is no prior published method for our task). For the baselines, we used both an SVM classifier as well as the C4.5 decision tree algorithm [36]. We have chosen to use a decision tree for the baselines as it can be linearized into decision rules, which may reflect the decisions taken manually by a biocurator who wishes to assess the correctness of relations. We argue that a decision-tree algorithm represents the closest algorithmic analog to a human biocurator who is assessing relations. We used different co-mention-based features to train several decision trees as follows:

- **Baseline 1:** Trained using document support values. The idea is that the higher the document support value, the higher the probability that the relationship is correct.
- **Baseline 2:** Trained using mutual information (MI) values based on co-mention of the two objects involved in a relation *I*(*o*_1_;*o*_2_). We recall that MI is a measure of the mutual dependence between the two variables.
- **Baseline 3:** Trained using document support values computed for each document field (three features are used). This baseline can be seen as a more elaborated version of Baseline 1.
- **Baseline 4:** Trained using mutual information (MI) values computed for each document field (three features are used). This baseline can be seen as a more elaborated version of Baseline 2.
- **WordEmb:** This baseline method relies on word embeddings,^12^ which may effectively allow consideration of indirect relations between pairs of objects. We developed several features extracted from the embeddings, including vector dot products and element-wise multiplication.

**Fig. 8:**
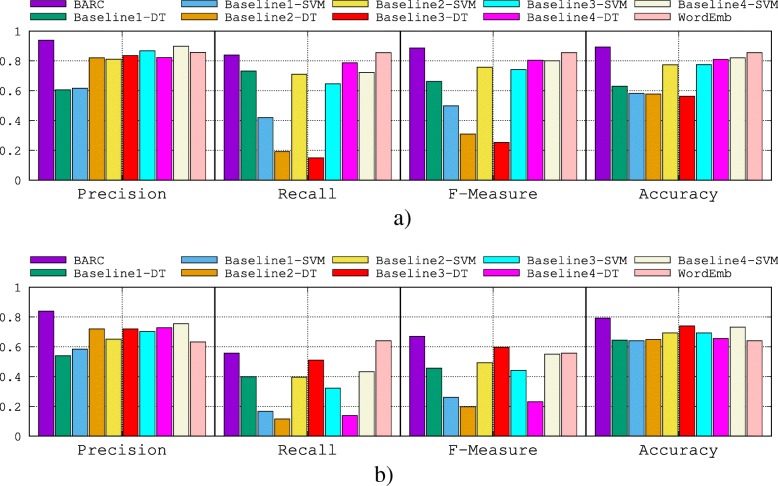
Performance comparison of BARC for *k*=30. DT: Decision Tree-based classification. **a** Performance comparison on gene–disease relations. **b** Performance comparison on protein–protein interactions

Results are shown in Fig. 8a and b for the gene–disease relations and the protein–protein interactions, respectively. Comparing the baselines, we first observe that the best results are those based on SVM as we may expect. This is because SVM tries to maximize the margin between the closest support vectors while Decision Trees maximize the information gain. Thus, SVM finds a solution which separates the two categories as far as possible while Decision Trees do not have this capability. BARC clearly outperforms the baselines on the two test sets. We also note that for the two test sets, the performance of BARC is stable compared to the other baselines; for example, Baseline 4 has a high recall value on gene–disease relations and thus is ranked second on that test set, but a low recall value on the protein–protein interactions and thus is ranked fourth on that test set. Finally, we observe that the word-embedding baseline is the best performing baseline on the gene-disease relations, and nearly as effective as BARC (which achieves only 3.5% improvement in F-Measure over the word embedding baseline). However, the word-embedding baseline is much less effective than BARC on the protein-protein interaction dataset. After a thorough investigation, we find that, compared to all baselines over the two datasets, the word-embedding approach has stable performance between the two tasks. However, BARC achieves higher performance on the protein–protein interactions dataset than on the gene–disease interactions dataset. We explain this primarily by the fact that the tool that we used to identify genes and proteins in the literature has a higher accuracy than the tool we used to identify diseases. Therefore, BARC could more accurately capture the context of the protein–protein interactions to correctly classify the corresponding relations.

## Discussion

In this section, we first provides a discussion on the informativeness of features and then we discuss the limitations of our method.

### Features analysis

To explore the relationship between features and the relation consistency labels, we undertook a feature analysis. Specifically, we performed a mutual information analysis at the level of individual features and feature ablation analysis at the level of feature category.

#### Mutual information analysis

**Fig. 9:**
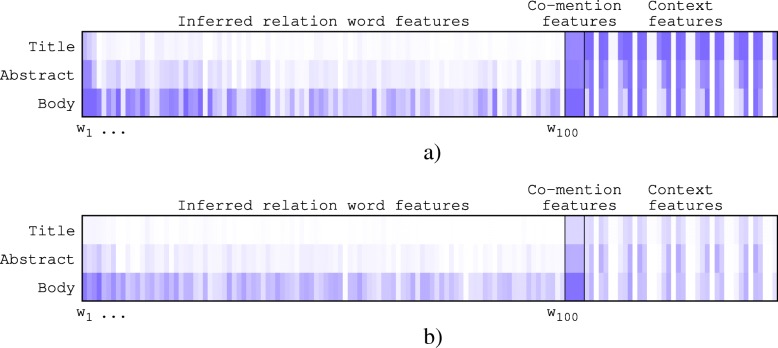
Feature analysis using MI. The higher the density color, the higher the MI value. **a** Gene-Disease relations. **b** Protein-Protein interactions

A general method for measuring the amount of information that a feature *x*_*k*_ provides with respect to predicting a class label *y* (“correct” or “incorrect”) is to calculate its mutual information (MI) *I*(*x*_*k*_,*y*). In Table 5, we present the list of 10 top-ranked features, and in Fig. 9, we show a complete overview of MI values obtained for each feature using a heatmap.

**Table 5:** Ranking of the most important features using MI

Rank	Type	Field	Feature
	Gene-disease relations		
1	Dict.	Body	Mutation
2	Dict.	Body	Gene
3	Co-men.	Body	Jaccard
4	Co-men.	Body	Dice
5	Co-men.	Body	Cosine
6	Context	Title	SumOverlap
7	Context	Title	SumJaccard
8	Context	Title	SumCosine
9	Context	Title	SumDice
10	Context	Title	SumMatching
	Protein-protein interactions		
1	Dict.	Body	Interact
2	Co-men.	Body	Overlap
3	Co-men.	Body	Cosine
4	Co-men.	Body	Jaccard
5	Co-men.	Body	Dice
6	Dict.	Body	Protein
7	Dict.	Body	Complex
8	Dict.	Body	Bind
9	Dict.	Body	Cell
10	Dict.	Body	Figur

This analysis led us to make the following observations. On both relations, the top features are the inferred relation words. Specifically, as we may expect, the words “mutation” and “interact” are the most discriminative for respectively gene–disease relations and protein–protein interactions. However, based on Fig. 9, all feature kinds are informative, as well as all document fields. Features computed on the body of documents are the most informative, particularly for inferred relation word and co-mention based features. For context-based features on the gene–disease relations, the titles of documents seem to be more informative than the other fields. This indicates that correct gene–diseases relations tend to be mentioned in the title of articles.

Given the large number of features and similarity metrics we have defined, we show in Table 6 the correlation between similar features for the different types of features using PCA. This table shows the most related and independent features. Also, this analysis shows which feature inside each feature group is most important. Based on the results we obtained, we make the following observations: correlated features come usually from similar fields of documents; all documents fields are useful for extraction of informative features; similar aggregation functions generate correlated features.

**Table 6:** Top correlated features within each group of features obtained using PCA

	Dictionary		Co-mention		Context	
	Field	Feature	Field	Feature	Field	Feature
	Gene-disease relations					
1	Body	study	Abstract	jaccard	Abstract	MeanOverlap
	Body	found	Abstract	dice	Abstract	MaxCosine
	Body	analysis	Abstract	cosine	Abstract	MeanMatching
	Body	report	Abstract	matching	Abstract	MeanDice
	Body	result	Body	cosine	Body	Coeff.VarJaccard
2	Body	susceptible	Title	matching	Title	Geo.MeanJaccard
	Body	associated	Title	jaccard	Title	Harm.MeanDice
	Abstract	susceptible	Title	dice	Title	Harm.MeanCosine
	Body	risk	Title	cosine	Title	Harm.MeanMatching
	Abstract	associated	Body	matching	Title	Harm.MeanJaccard
3	Body	cell	Abstract	cosine	Abstract	Harm.MeanCosine
	Body	interact	Abstract	dice	Abstract	Geo.MeanCosine
	Body	complex	Abstract	jaccard	Abstract	MinMatching
	Body	bind	Body	dice	Abstract	Geo.MeanDice
	Body	figure	Body	jaccard	Abstract	Geo.MeanJaccard
	Protein-protein interaction					
1	Body	result	Abstract	Dice	Title	MaxCosine
	Body	study	Abstract	Cosine	Title	MaxDice
	Body	cell	Abstract	Matching	Title	MaxOverlap
	Body	show	Body	Jaccard	Title	MaxJaccard
	Body	shown	Body	Dice	Title	MaxMatching
2	Abstract	express	Title	Jaccard	Abstract	Harm.MeanDice
	Body	yeast	Title	Dice	Abstract	Harm.MeanCosine
	Body	termin	Title	Cosine	Abstract	Geo.MeanJaccard
	Abstract	receptore	Title	Matching	Abstract	Geo.MeanDice
	Body	complex	Body	Cosine	Abstract	Geo.MeanCosine
3	Title	residue	Abstract	Matching	Title	Geo.MeanDice
	Title	receptore	Title	Matching	Title	Harm.MeanCosine
	Title	hla	Body	Matching	Title	MinCosine
	Body	hla	Title	Jaccard	Title	Harm.MeanMatching
	Abstract	hla	Body	Dice	Title	MinDice

#### Feature ablation analysis

**Fig. 10:**
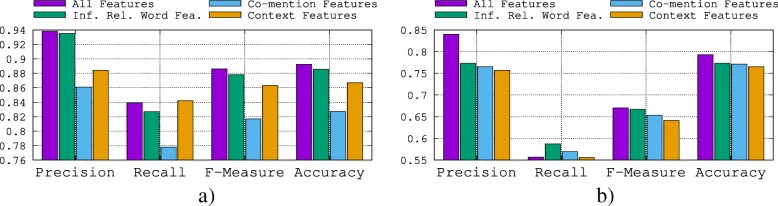
Feature ablation analysis (*k*=30). **a** Performance on gene–disease relations. **b** Performance on protein–protein interactions

A feature ablation study typically refers to removing some “feature group” of the model, and observing how that affects performance. Hence, we propose to study the impact of each feature group individually on the global performance. The results obtained are shown in Fig. 10 for both gene–disease relations and protein–protein interactions.

We make the following observations: For gene–disease relations, the best feature groups are in the following order: inferred relation word features followed by context features and then by co-mention features. For the protein–protein interactions, the best feature groups are in the following order: inferred relation word features followed by co-mention features and then by context features. On the protein–protein interactions, all features lead to similar performance, yielding only marginal improvements on each other. The combination of the three feature groups on the gene–disease relations is producing marginal improvements. However, their combination slightly improves the performance on the protein–protein interactions, boosting precision from 76% for inferred relation word features to 84% (an improvement of roughly 10.5%).

### Limitations

There are limitations in our approach. Currently, BARC incorrectly identifies negative sentences, expressing that two objects do *not* interact in a given relation, as positive instances of their interaction. For example, given a document that contains a sentence such as: *“Gene X does not affect the expression in Syndrome Y”*, may count as a support of the relation *(geneX, affect, SyndromeY)*. However, given the positivity bias of research publications, we expect such negative statements to be outweighed by positive statements of true interactions. The issue could be addressed by explicit treatment of negation, either through a naïve approach of removing sentences containing negation key terms, or through analysis of sentence structure to identify negated relations, for instance as done in the BioNLP Shared Task [37].

Second, BARC relies on the capability and accuracy of named-entity-recognition tools to extract object mentions from the literature. Even though the tools we have used to extract gene and disease mentions from the literature have reasonable accuracy, we assumed in this work that they are highly reliable. Thus, we have not considered the impact of their errors on the results that we have reported.

Finally, BARC also relies on the fact that valid and correct relationships are expected to occur in the same documents and sentences. Indeed, if a given relationship between two entities is supported by the literature, they should be discussed in individual documents, and moreover in the same sentences, to be correctly classified by BARC. Conversely, a valid relationship that is not directly stated in the literature will not be correctly classified by BARC. This could be addressed by enhancing BARC with external sources of knowledge.

## Related work

There is a substantial body of research related to named entity recognition, relation extraction, and the use of structured or unstructured knowledge for checking statements and answering questions. Hence, we review below the major works related to these aspects.

### Named entity recognition (NER)

Recognition of named entities and concepts in text, such as genes and diseases, is the basis for most biomedical applications of text mining [38]. NER is sometimes divided into two subtasks: *recognition*, which consists of identifying words of interest, and *normalization*, which consists of mapping the identified entities to the correct entries in a dictionary. However, as noted by Pletscher-Frankild et al. [34], because recognition without normalization has limited practical use the normalization step is now often implicitly considered part of the NER task.

In brief, the main issues with NER in the biological literature occur because of the poor standardization of names and the fact that a name of an entity may have other meanings [39]. To recognize names in text, many systems make use of rules that look at features of names themselves, such as capitalization, word endings and the presence of numbers, as well as contextual information from nearby words. In early methods, rules were hand-crafted [40], whereas newer methods make use of machine learning and deep learning techniques [41, 42], relying on the availability of manually annotated training datasets.

Dictionary-based methods instead rely on matching a dictionary of names against text. For this purpose the quality of the dictionary is obviously very important; the best performing methods for NER according to blind assessments rely on carefully curated dictionaries to eliminate synonyms that give rise to many false positives [43, 44]. Moreover, dictionary-based methods have the crucial advantage of being able to normalize names. Whether or not use is made of machine learning, a high-quality, comprehensive dictionary of gene and disease names is a prerequisite for mining disease–gene associations from the biomedical literature.

### Relation extraction

Relation extraction is the task of extracting semantic relationships between named entities mentioned in a text. Relation extraction methods are usually divided into two categories [45, 46], namely: feature-based and kernel-based supervised learning approaches. In feature-based methods, for each relation instance in the labelled data, a set of features is generated and a classifier is then trained to classify any new relation instance [47–49]. On the other hand, kernel based methods use kernel functions to compute similarities between representations of two relation instances and SVM is employed for classification. Two major kernel approaches are used: bag-of-features kernels [50, 51] and tree or graph kernels [52–54, 54–56].

With the rise of deep learning, several neural network models have been used for relation extraction while achieving obtained state-of-the-art performance. Two major neural architectures were used including convolutional neural networks (CNNs) [57–63] and recurrent neural networks (RNNs) [64–67]. Combinations of these two architectures have also been proposed [68–70].

### Methods for assessing statements

Identifying correct and incorrect statements is a problem that has been widely explored in the literature using various techniques ranging from pure information retrieval to crowdsourcing, and used in many applications such as fact-checking in journalism and the support of biomedical evidence from the literature. We review below the major work related to our paper.

#### Fact-checking

Several approaches have been recently proposed to check the truthfulness of facts, particularly in the context of journalism [71]. Most of the methods proposed assume the availability of structured knowledge modeled as a graph, which is then used to check the consistency of facts. Hence, recent works addressed this problem as a graph search problem [72, 73], a link prediction problem [74], or a classification and ranking problem [75].

However, the aforementioned methods rely on the availability of a knowledge graph, which limits and reduces the scope and the impact of any method as: it requires a specific knowledge graph for each domain (eventually sub-domain), and may fail in real-time assessment of facts, as it requires continuous update of the knowledge graph, a task generally done by domain-specialists. BARC may overcome these two limitations as it uses a standard index, which can easily be updated to address new facts.

#### Supporting biomedical evidence

In the context of biocuration, the information retrieval task that consists of finding relevant articles for the curation of a biological database is called *triage* [76–80]. Triage can be seen as a *semi-automated* method for assessing biological statements; it assists curators in the selection of relevant articles, which then must be manually reviewed to decide their correctness. As mentioned in the Introduction, this triage process is time-consuming and expensive [4, 5]; curation of a single protein may take up to a week and requires considerable human investment both in terms of knowledge and effort [6]. Rather, BARC is a fully automated method that directly helps biocurators to assess biological database assertions using the scientific literature.

Also, in the biomedical context, Light et al. [81] used a handcrafted list of cues to identify speculative sentences in MEDLINE abstract by looking for specific keywords which imply speculative content. However, it is unclear how this method generalizes to relations that are mentioned in both speculative and non-speculative sentences. Leach et al. [82] proposed a knowledge-based system that combines reading, reasoning, and reporting methods to facilitate analysis of experimental data, which was then applied to the analysis of a large-scale gene expression array data sets relevant to craniofacial development. This method combines and integrates multiple sources of data into a knowledge network through a reading and a reasoning component; as it requires complex mappings to identify and link entities in order to get a consistent knowledge graph, it may fail to scale to large datasets. Zerva et al. [83] proposed the use of uncertainty as an additional measure of confidence for interactions in biomedical pathway network supported by evidence from literature for model curation. The method is based on using a hybrid approach that combines rule induction and machine learning. This method focuses on biomedical pathways and uses features specifically designed to detect uncertain interactions. However, its generalisation for assessment other statements is unclear and is not discussed in the paper. Several other papers [84, 85] have focused on extracting relations from text using distant supervision multi-instance learning to reduce the amount of manual effort for labeling. Such work demonstrates that the approach can be successfully used to extract relations from literature about a biological process with little or no manually annotated corpus data. This method might be complementary to BARC; it may allow the extraction of new relevant features that characterize the relationships between entities. Note that BARC is capable of overcoming all the drawbacks mentioned for these approaches, as it does not require any data integration or complex mappings and it has been designed in such a way to assess multiple types of statements.

#### Crowdsourcing-based methods

Crowdsourcing has attracted interest in the bioinformatics domain for data annotation [86]. It has been successfully applied in the biomedical and clinical domains [87]. This research has demonstrated that crowdsourcing is an inexpensive, fast, and practical approach for collecting high quality annotations for different BioIE tasks [88], including NER in clinical trial documents [89], disease mention annotation in PUBMED literature [90], relation extraction between clinical problems and medications [91], etc. Different techniques have been explored to improve the quality and effectiveness of crowdsourcing, including probabilistic reasoning [92] to make sensible decisions on annotation candidates and gamification strategies [93] to motivate the continuous involvement of the expert crowd. More recently, a method called CrowdTruth [94] was proposed for collecting medical ground truth through crowdsourcing, based on the observation that disagreement analysis on crowd annotations can compensate lack of medical expertise of the crowd. Experiments with the use of CrowdTruth for a medical relation extraction task show that the crowd performs just as well as medical experts in terms of quality and efficacy of annotation, and also indicate that at least 10 workers per sentence are needed to get the highest quality annotation for this task. Crowdsourcing holds promise for biocuration tasks as well and could be combined with prioritisation methods such as BARC provides.

#### Information retrieval-based methods

In the context of information retrieval, despite the development of sophisticated techniques for helping users to express their needs, many queries still remain unanswered [95]. Hence, to tackle these issues, other types of question-answering (QA) systems have emerged to allow people to help each other to answer questions [96]. Community QA systems provide a means for answering several types of questions such as recommendation, opinion seeking, factual knowledge, or problem solving [96]. (Therefore, QA systems can help to answer specific and contextualised questions, and hence, could potentially be used by biocurators for seeking answers to questions like those given as examples in “Problem definition” section.

A challenge facing such systems is in the response time as well as the quality of the answers [95, 97–99]. However, the time factor is critical in the context of biocuration as there is a huge quantity of information to be curated. Such waiting times to answer questions probably disqualify this body of work for their usage in biocuration.

These methods that use unstructured knowledge (that is, the literature) for fact-checking and query answering address the problem as a pure information retrieval problem. In other words, these methods rank documents (or sentences) relevant to a given information need, and it is up to the user to read and search for a support to a given assertion or question. In contrast, BARC allows spotting of potentially incorrect assertions, and presents them to the user with, in our experiments, a high classification accuracy.

## Conclusions

We have described BARC, a tool that aims to help biocurators in checking the correctness of biological relations using the scientific literature. Specifically, given a new biological assertion represented as a relation, BARC first retrieves a list of documents that may be used to check the correctness of that relation. This retrieval step is done using a set-based retrieval algorithm (SaBRA). This list of documents is aggregated in order to compute features for the relation as a whole, which are used for a prediction task.

We evaluated BARC and retrieval algorithm SaBRA using publicly available datasets including the PubMed Central collection and two types of relational assertions, gene–disease relations, and protein–protein interactions. The results obtained showed that BARC substantially outperforms the best baselines, with an improvement of F-measure of 3.5% and 13%, respectively, on gene-disease relations and protein-protein interactions. We have additionally carried out a feature analysis that showed that all feature types are informative, as are all fields of the documents. A limitation of this work is that it relies on the accuracy of the GNormPlus [31] and DNorm [32] entity recognition tools; these are automated tools and hence subject to error. We note further that in this paper BARC has been evaluated on only two kinds of relations; we leave the analysis of its generalization to future work, such as drug-disease or drug-drug interactions. However, the results show that the methods are effective enough to be used in practice, and we believe they can provide a valuable tool for supporting biocurators.

## Abbreviations

BARC: Biocuration tool for assessment of relation consistency
CTD: Comparative toxicogenomics database
FP: False positive
PPI: Protein-protein interaction
RBF: Radial basis function
ROC: Receiver operating characteristic
SaBRA: Set-based retrieval algorithm
SVM: Support vector machine
TF-IDF: Term frequency-inverse document frequency
TP: True positive

## Footnotes

1
https://www.ncbi.nlm.nih.gov/genbank/

2
http://www.uniprot.org/

3
http://www.ncbi.nlm.nih.gov/pubmed/

4
https://github.com/rbouadjenek/DQBioinformatics

5 Atopy refers to the genetic tendency to develop allergic diseases such as allergic rhinitis, asthma and atopic dermatitis (eczema).

6 http://www.ncbi.nlm.nih.gov/pmc/tools/openftlist/, downloaded October 2015.

7
https://figshare.com/articles/DISEASES_v1/1367457

8 https://thebiogrid.org/download.php(Release 3.4.150)

9
http://www.ncbi.nlm.nih.gov/pmc/articles/PMC100320/

**Table 4:** Dataset statistics

Articles statistics					
	Mentions				
# articles	Mention	Avg	Max	Max entity	
1,135,611	Gene	6.15	2040	PMC100320	
	Disease	11.01	1272	PMC100785	
Object mentions in #documents					
Mention	# uniq men.	Avg	Max	Max entity	
Gene	54,447,840	24.56	61,248	NAT2	
Disease	55,850,078	1402	273,007	Heavy chain	
Relational statements					
Type	Avg	Min	Max	Correct	Incorrect
Etiology	141.86	1	12,296	989	1002
PPIs	14.11	1	1626	1758	2899

10
https://www.ncbi.nlm.nih.gov/gene/?term=10

11 We used the Lucene implementation of TF-IDF and Okapi BM25, https://lucene.apache.org/.

12 We used word vectors induced from PMC by Pyysalo et al. [100]: http://bio.nlplab.org/

## References

[1] Baxevanis AD, Bateman A (2015). The importance of biological databases in biological discovery. Curr Protocol Bioinforma.

[2] Bateman A (2010). Curators of the world unite: the international society of biocuration. Bioinformatics.

[3] NCBI Resource Coordinators (2017). Database resources of the national center for biotechnology information. Nucleic Acids Res.

[4] Poux S, Magrane M, Arighi CN, Bridge A, O’Donovan C, Laiho K, The UniProt Consortium (2014). Expert curation in UniProtKB: a case study on dealing with conflicting and erroneous data. Database.

[5] The UniProt Consortium (2017). UniProt: the universal protein knowledgebase. Nucleic Acids Res.

[6] Poux S, Arighi CN, Magrane M, Bateman A, Wei C-H, Zhiyong L, Boutet E, Bye-A-Jee H, Famiglietti ML, Roechert B, The UniProt Consortium (2017). On expert curation and scalability: UniProtKB/Swiss-Prot as a case study. Bioinformatics.

[7] Zou D, Ma L, Jun Y, Zhang Z (2015). Biological databases for human research. Genom Proteomics Bioinforma.

[8] Koh JLY, Lee ML, Brusic V. A classification of biological data artifacts. In: Workshop on Database Issues in Biological Databases: 2005. p. 53–7.

[9] Chen Q, Zobel J, Verspoor K (2017). Duplicates, redundancies and inconsistencies in the primary nucleotide databases: a descriptive study. Database.

[10] Baumgartner Jr. WA, K. Bretonnel C, Fox L, Acquaah-Mensah GK, Hunter L (2007). Manual curation is not sufficient for annotation of genomic databases. Bioinformatics.

[11] Helmy M, Crits-Christoph A, Bader GD (2016). Ten simple rules for developing public biological databases. PLoS Comput Biol.

[12] Bouadjenek MR, Verspoor K, Zobel J (2017). Automated detection of records in biological sequence databases that are inconsistent with the literature. J Biomed Inform.

[13] Bouadjenek MR, Verspoor K, Zobel J (2017). Literature consistency of bioinformatics sequence databases is effective for assessing record quality. Database.

[14] Bouadjenek MR, Verspoor K, Zobel J (2017). Learning biological sequence types using the literature. Proceedings of the 26th ACM Conference on Information and Knowledge Management, CIKM ’17.

[15] Brookes AJ, Robinson PN (2015). Human genotype-phenotype databases: aims, challenges and opportunities. Nat Rev Genet.

[16] Sigrist CJA, Cerutti L, De Castro E, Langendijk-Genevaux PS, Bulliard V, Bairoch A, Hulo N (2009). Prosite, a protein domain database for functional characterization and annotation. Nucleic Acids Res.

[17] Benson ML, Smith RD, Khazanov NA, Dimcheff B, Beaver J, Dresslar P, Nerothin J, Carlson HA (2007). Binding moad, a high-quality protein–ligand database. Nucleic Acids Res.

[18] Mering CV, Krause R, Snel B, Cornell M, Oliver SG (2002). Stanley Fields, and Peer Bork. Comparative assessment of large-scale data sets of protein-protein interactions. Nature.

[19] Hu G, Agarwal P (2009). Human disease-drug network based on genomic expression profiles. PLoS ONE.

[20] Wiegers TC, Davis AP, Cohen KB, Hirschman L, Mattingly CJ (2009). Text mining and manual curation of chemical-gene-disease networks for the Comparative Toxicogenomics Database (CTD). BMC Bioinformatics.

[21] Salton G, Wong A, Yang CS (1975). A Vector Space Model for Automatic Indexing. Commun ACM.

[22] Robertson SE, Walker S, Jones S, Hancock-Beaulieu M, Gatford M (1993). Okapi at trec-2. TREC.

[23] Singhal A, Buckley C, Mitra M (1996). Pivoted document length normalization. Proceedings of the 19th Annual International ACM SIGIR Conference on Research and Development in Information Retrieval, SIGIR ’96.

[24] Bunescu R, Mooney R, Ramani A, Marcotte E (2006). Integrating co-occurrence statistics with information extraction for robust retrieval of protein interactions from medline. Proceedings of the workshop on linking natural language processing and biology: towards deeper biological literature analysis.

[25] Pyysalo S, Airola A, Heimonen J, Björne J, Ginter F, Salakoski T (2008). Comparative analysis of five protein-protein interaction corpora. BMC Bioinformatics.

[26] Markines B, Cattuto C, Menczer F, Benz D, Hotho A, Stumme G (2009). Evaluating similarity measures for emergent semantics of social tagging. Proceedings of the 18th International Conference on World Wide Web, WWW ’09.

[27] Wang X, Zhai C (2008). Mining term association patterns from search logs for effective query reformulation. Proceedings of the 17th ACM Conference on Information and Knowledge Management, CIKM ’08.

[28] Zhai C, Lafferty J (2001). A study of smoothing methods for language models applied to ad hoc information retrieval. Proceedings of the 24th Annual International ACM SIGIR Conference on Research and Developmentz in Information Retrieval, SIGIR ’01.

[29] Cortes C, Vapnik V (1995). Support-vector networks. Mach Learn.

[30] Chang C-C, Lin C-J (2011). Libsvm: A library for support vector machines. ACM Trans Intell Syst Technol.

[31] Wei C-H, Kao H-Y, Lu Z (2015). GNormPlus: An integrative approach for tagging genes, gene families, and protein domains. BioMed Res Int.

[32] Leaman R, Dogan RI, Lu Z (2013). DNorm: disease name normalization with pairwise learning to rank. Bioinformatics.

[33] Bouadjenek MR, Verspoor K (2017). Multi-field query expansion is effective for biomedical dataset retrieval. Database.

[34] Pletscher-Frankild S, Palleja A, Tsafou K, Binder JX, Jensen LJ (2015). Diseases: Text mining and data integration of disease-gene associations. Methods.

[35] Stark C, Breitkreutz B-J, Reguly T, Boucher L, Breitkreutz A, Tyers M (2006). BioGRID: a general repository for interaction datasets. Nucleic Acids Res.

[36] Quinlan JR (1993). C4.5: Programs for Machine Learning.

[37] Kim J-D, Ohta T, Pyysalo S, Kano Y, Tsujii J. Overview of bionlp’09 shared task on event extraction. In: Proceedings of the Workshop on Current Trends in Biomedical Natural Language Processing: Shared Task. Association for Computational Linguistics: 2009. p. 1–9.

[38] Jensen LJ, Saric J, Bork P (2006). Literature mining for the biologist: from information retrieval to biological discovery. Nat Rev Genet.

[39] Chen L, Liu H, Friedman C (2005). Gene name ambiguity of eukaryotic nomenclatures. Bioinformatics.

[40] Fukuda K, Tsunoda T, Tamura A, Takagi T, et al.Toward information extraction: identifying protein names from biological papers. In: Pac symp biocomput, vol. 707: 1998. p. 707–18. 9697224

[41] Zhou G, Shen D, Zhang J, Jian S, Tan S (2005). Recognition of protein/gene names from text using an ensemble of classifiers. BMC Bioinformatics.

[42] Settles B (2005). Abner: an open source tool for automatically tagging genes, proteins and other entity names in text. Bioinformatics.

[43] Hanisch D, Fundel K, Mevissen H-T, Zimmer R, Fluck J (2005). Prominer: rule-based protein and gene entity recognition. BMC Bioinformatics.

[44] Gaudan S, Kirsch H, Rebholz-Schuhmann D (2005). Resolving abbreviations to their senses in medline. Bioinformatics.

[45] Pawar S, Palshikar GK, Bhattacharyya P. Relation Extraction: A Survey. ArXiv e-prints. 2017.

[46] Bach N, Badaskar S. A review of relation extraction. Technical report: Carnegie Mellon University; 2007.

[47] Kambhatla N. Combining lexical, syntactic, and semantic features with maximum entropy models for extracting relations. In: Proceedings of the ACL 2004 on Interactive Poster and Demonstration Sessions, ACLdemo ’04. Association for Computational Linguistics: 2004.

[48] GuoDong Z, Jian S, Jie Z, Min Z. Exploring various knowledge in relation extraction. In: Proceedings of the 43rd Annual Meeting on Association for Computational Linguistics, ACL ’05. Association for Computational Linguistics: 2005. p. 427–34.

[49] Zhao S, Grishman R (2005). Extracting relations with integrated information using kernel methods. Proceedings of the 43rd Annual Meeting on Association for Computational Linguistics, ACL ’05.

[50] McDonald R, Pereira F, Kulick S, Winters S, Jin Y, White P (2005). Simple algorithms for complex relation extraction with applications to biomedical ie. Proceedings of the 43rd Annual Meeting on Association for Computational Linguistics, ACL ’05.

[51] Bunescu RC, Mooney RJ (2005). Subsequence kernels for relation extraction. Proceedings of the 18th International Conference on Neural Information Processing Systems, NIPS’05.

[52] Collins M, Duffy N (2001). Convolution kernels for natural language. Proceedings of the 14th International Conference on Neural Information Processing Systems: Natural and Synthetic, NIPS’01.

[53] Zelenko D, Aone C, Richardella A (2003). Kernel methods for relation extraction. J Mach Learn Res.

[54] Panyam NC, Verspoor K, Cohn T, Ramamohanarao K (2018). Exploiting graph kernels for high performance biomedical relation extraction. J Biomed Semant.

[55] Panyam NC, Verspoor K, Cohn T, Kotagiri R. Asm kernel: Graph kernel using approximate subgraph matching for relation extraction. In: Proceedings of the Australasian Language Technology Association Workshop 2016: 2016. p. 65–73.

[56] Panyam NC, Verspoor K, Cohn T, Ramamohanarao K. Exploiting tree kernels for high performance chemical induced disease relation extraction. In: Proceedings of the 7th International Symposium on Semantic Mining in Biomedicine. BioMed Central: 2016. p. 4–5.

[57] Zeng D, Liu K, Lai S, Zhou G, Zhao J (2014). Relation classification via convolutional deep neural network. COLING 2014, 25th International Conference on Computational Linguistics, Proceedings of the Conference: Technical Papers, August 23-29, 2014, Dublin, Ireland.

[58] Nguyen TH, Grishman R. Relation extraction: Perspective from convolutional neural networks. In: Proceedings of the 1st Workshop on Vector Space Modeling for Natural Language Processing, VS@NAACL-HLT 2015, June 5, 2015, Denver, Colorado, USA. Association for Computational Linguistics: 2015. p. 39–48.

[59] Zeng D, Liu K, Chen Y, Zhao J. Distant supervision for relation extraction via piecewise convolutional neural networks. In: Proceedings of the 2015 Conference on Empirical Methods in Natural Language Processing. Association for Computational Linguistics: 2015. p. 1753–62.

[60] Lin Y, Shen S, Liu Z, Luan H, Sun M. Neural relation extraction with selective attention over instances. In: Proceedings of the 54th Annual Meeting of the Association for Computational Linguistics (Volume 1: Long Papers), vol. 1. Association for Computational Linguistics: 2016. p. 2124–33.

[61] Jiang X, Wang Q, Li P, Wang B. Relation extraction with multi-instance multi-label convolutional neural networks. In: Proceedings of COLING 2016, the 26th International Conference on Computational Linguistics: Technical Papers. The COLING 2016 Organizing Committee: 2016. p. 1471–80.

[62] Zeng W, Lin Y, Liu Z, Sun M. Incorporating relation paths in neural relation extraction. In: Proceedings of the 2017 Conference on Empirical Methods in Natural Language Processing, EMNLP 2017, Copenhagen, Denmark, September 9-11, 2017. Association for Computational Linguistics: 2017. p. 1768–77.

[63] Huang Y, Wang WY (2017). Deep residual learning for weakly-supervised relation extraction. Proceedings of the 2017 Conference on Empirical Methods in Natural Language Processing (EMNLP 2017), Copenhagen.

[64] Miwa M, Bansal M. End-to-end relation extraction using lstms on sequences and tree structures. In: Proceedings of the 54th Annual Meeting of the Association for Computational Linguistics (Volume 1: Long Papers), vol. 1: 2016. p. 1105–16.

[65] Zhang M, Zhang Y, Fu G. End-to-end neural relation extraction with global optimization. In: Proceedings of the 2017 Conference on Empirical Methods in Natural Language Processing: 2017. p. 1730–40.

[66] Katiyar A, Cardie C. Going out on a limb: Joint extraction of entity mentions and relations without dependency trees. In: Proceedings of the 55th Annual Meeting of the Association for Computational Linguistics (Volume 1: Long Papers), vol. 1: 2017. p. 917–28.

[67] Ammar W, Peters M, Bhagavatula C, Power R. The ai2 system at semeval-2017 task 10 (scienceie): semi-supervised end-to-end entity and relation extraction. In: Proceedings of the 11th International Workshop on Semantic Evaluation (SemEval-2017): 2017. p. 592–6.

[68] Nguyen TH, Grishman R. Combining neural networks and log-linear models to improve relation extraction. In: Proceedings of IJCAI Workshop on Deep Learning for Artificial Intelligence: 2016.

[69] Raj D, Sahu S, Anand A. Learning local and global contexts using a convolutional recurrent network model for relation classification in biomedical text. In: Proceedings of the 21st Conference on Computational Natural Language Learning (CoNLL 2017): 2017. p. 311–21.

[70] Nguyen DQ, Verspoor K. Convolutional neural networks for chemical-disease relation extraction are improved with character-based word embeddings. In: Proceedings of the BioNLP 2018 workshop, Melbourne, Australia, July 19, 2018: 2018. p. 129–36.

[71] Vlachos A, Riedel S. Fact checking: Task definition and dataset construction. In: ACL 2014: 2014. p. 18.

[72] Ciampaglia GL, Shiralkar P, Rocha LM, Bollen J, Menczer F, Flammini A (2015). Computational fact checking from knowledge networks. PLoS ONE.

[73] Shiralkar P, Flammini A, Menczer F, Ciampaglia GL. Finding Streams in Knowledge Graphs to Support Fact Checking. ArXiv e-prints. 2017.

[74] Shi B, Weninger T (2016). Discriminative predicate path mining for fact checking in knowledge graphs. Knowl-Based Syst.

[75] Hassan N, Arslan F, Li C, Tremayne M (2017). Toward automated fact-checking: Detecting check-worthy factual claims by claimbuster. Proceedings of the 23rd ACM SIGKDD International Conference on Knowledge Discovery and Data Mining, KDD ’17.

[76] Valencia A, Mattingly C, Arighi CN, Cohen KB, Hirschman L, Krallinger M, Wiegers TC, Wilbur WJ, Lu Z, Wu CH (2012). BioCreative-2012 Virtual Issue. Database.

[77] Wiegers TC, Davis AP, Mattingly CJ (2012). Collaborative biocuration–text-mining development task for document prioritization for curation. Database.

[78] Mottin L, Pasche E, Gobeill J, de Laval VR, Gleizes A, Michel P-A, Bairoch A, Gaudet P, Ruch P (2017). Triage by ranking to support the curation of protein interactions. Database.

[79] Roechert B, Boutet E, Famiglietti ML, Poux S (2017). The UniProt Consortium, Cecilia N Arighi, Alex Bateman, Hema Bye-A-Jee, Michele Magrane, Chih-Hsuan Wei, and Zhiyong Lu. On expert curation and scalability: UniProtKB/Swiss-Prot as a case study. Bioinformatics.

[80] Chen Q, Panyam NC, Elangovan A, Verspoor K (2018). BioCreative VI Precision Medicine Track system performance is constrained by entity recognition and variations in corpus characteristics. Database.

[81] Light M, Qiu XY, Srinivasan P (2004). The language of bioscience: Facts, speculations, and statements in between. Lynette Hirschman and James Pustejovsky, editors, HLT-NAACL 2004 Workshop: BioLINK 2004, Linking Biological Literature, Ontologies and Databases.

[82] Leach SM, Tipney H, Feng W, Baumgartner Jr. WA, Kasliwal P, Schuyler RP, Williams T, Spritz RA, Hunter L (2009). Biomedical discovery acceleration, with applications to craniofacial development. PLoS Comput Biol.

[83] Zerva C, Batista-Navarro RT, Day P, Ananiadou S. Using uncertainty to link and rank evidence from biomedical literature for model curation. Bioinformatics. 2017; 3:7. 10.1093/bioinformatics/btx466 PMC5860317 29036627

[84] Ravikumar KE, Liu H, Cohn JD, Wall ME, Verspoor KM. Literature mining of protein-residue associations with graph rules learned through distant supervision. In: J. Biomedical Semantics: 2012. 10.1186/2041-1480-3-S3-S2 PMC3465209 23046792

[85] Lamurias A, Clarke LA, Couto FM (2017). Extracting microrna-gene relations from biomedical literature using distant supervision. PLoS ONE.

[86] Good BM, Su AI (2013). Crowdsourcing for bioinformatics. Bioinformatics.

[87] Khare R, Good BM, Leaman R, Su AI, Lu Z (2016). Crowdsourcing in biomedicine: challenges and opportunities. Brief Bioinform.

[88] Liu F, Chen J, Jagannatha A, Yu H. Learning for biomedical information extraction: Methodological review of recent advances. arXiv preprint arXiv:1606.07993. 2016.

[89] Zhai H, Lingren T, Deleger L, Qi L, Kaiser M, Stoutenborough L, Imre S (2013). Web 2.0-based crowdsourcing for high-quality gold standard development in clinical natural language processing. J Med Internet Res.

[90] Good BM, Nanis M, Wu C, Su AI. Microtask crowdsourcing for disease mention annotation in pubmed abstracts. In: Pacific Symposium on Biocomputing Co-Chairs: 2014. p. 282–93. World Scientific. PMC4299946 25592589

[91] McCoy AB, Wright A, Laxmisan A, Ottosen MJ, McCoy JA, Butten D, Sittig DF (2012). Development and evaluation of a crowdsourcing methodology for knowledge base construction: identifying relationships between clinical problems and medications. J Am Med Inform Assoc.

[92] Demartini G, Difallah DE, Cudré-Mauroux P (2012). Zencrowd: Leveraging probabilistic reasoning and crowdsourcing techniques for large-scale entity linking. Proceedings of the 21st International Conference on World Wide Web, WWW ’12.

[93] Dumitrache A, Aroyo L, Welty C, Sips R-J, Levas A (2013). “Dr. Detective”: Combining gamication techniques and crowdsourcing to create a gold standard in medical text. Proceedings of the 1st International Conference on Crowdsourcing the Semantic Web - Volume 1030, CrowdSem’13, pages 16–31, Aachen, Germany.

[94] Dumitrache A, Aroyo L, Welty CA. Achieving expert-level annotation quality with crowdtruth: The case of medical relation extraction. In: BDM2I@ISWC: 2015.

[95] Dror G, Koren Y, Maarek Y, Szpektor I. I want to answer; who has a question?: Yahoo! answers recommender system. In: Proceedings of the 17th ACM SIGKDD International Conference on Knowledge Discovery and Data Mining, KDD ’11. New York: ACM: 2011. p. 1109–17.

[96] Hoogeveen D, Wang L, Baldwin T, Verspoor KM (2018). Web forum retrieval and text analytics: A survey. Found Trends Inf Retr.

[97] Bouadjenek MR, Hacid H, Bouzeghoub M (2016). Social networks and information retrieval, how are they converging? a survey, a taxonomy and an analysis of social information retrieval approaches and platforms. Inf Syst.

[98] Hsieh G, Counts S (2009). mimir: a market-based real-time question and answer service. Proceedings of the SIGCHI Conference on Human Factors in Computing Systems, CHI ’09.

[99] Zhang J, Ackerman MS, Adamic L, Kyung K (2007). Nam. Qume: a mechanism to support expertise finding in online help-seeking communities. Proceedings of the 20th annual ACM symposium on User interface software and technology, UIST ’07.

[100] Moen SPFGH, Ananiadou TSS. Distributional semantics resources for biomedical text processing. In: Proceedings of the 5th International Symposium on Languages in Biology and Medicine, Tokyo, Japan: 2013. p. 39–43.

